# A study in dualism: The strange case of Dr. Jekyll and Mr. Hyde

**DOI:** 10.4103/0019-5545.43624

**Published:** 2008

**Authors:** Shubh M. Singh, Subho Chakrabarti

**Affiliations:** Department of Psychiatry, Post Graduate Institute of Medical Education and Research, Chandigarh - 160 012, India

**Keywords:** Dualism, literature, psychiatry

## Abstract

R. L. Stevenson's novel, The Strange Case of Dr. Jekyll and Mr. Hyde is a prominent example of Victorian fiction. The names Jekyll and Hyde have become synonymous with multiple personality disorder. This article seeks to examine the novel from the view point of dualism as a system of philosophy and as a religious framework and also from the view point of Freud's structural theory of the mind.

## DUALISM

Dualism derives from the Latin word *duo*, meaning two. Simply put, dualism can be understood as a thought that facts about the world in general or of a particular class cannot be explained except by supposing ultimately the existence of two different, often opposite, and irreducible principles. Dualism is most often discussed in context of the systems of religion and philosophy.[1]

The purpose of this paper is to examine Robert Stevenson's famous novel, “*The Strange Case of Dr. Jekyll and Mr. Hyde*”[2] from the view point of the above mentioned systems and to discuss the novel from a psychological perspective.

## THE AUTHOR AND THE NOVEL

Robert Balfour Louis Stevenson was a Scottish novelist, short story writer, and poet. Born in 1850, he was a qualified advocate but earned his living as a writer. He was chronically afflicted with tuberculosis, and dabbled with various psychotropic drugs such as alcohol, cannabis, and opium. He is well known for his dark and sinister tales like *Markheim, Thrawn Janet,* and racy adventure novels such as *Treasure Island* and *Kidnapped*. Successful and famous, he died at a young age in 1894. Interestingly enough, Stevenson later claimed that the plot of *The Strange Case of Dr Jekyll and Mr. Hyde* was revealed to him in a dream.[3]

*The Strange Case of Dr. Jekyll and Mr. Hyde* deals with a Dr. Henry Jekyll who is widely respected, successful, and possesses a brilliant intellect but is only too aware of the duplicity of the life that he leads, and of the evil that resides within him. Dr. Jekyll covertly provides utterance to the evil in his soul by various unspeakable acts, but is afraid of doing so openly because of the fear of social criticism. In the course of his experiments, he succeeds in producing a concoction that enables him to free this evil in him from the control of his good self, thus giving rise to Edward Hyde. Edward Hyde is pure evil and amoral. Not only is his psyche different from Dr. Jekyll but also his body is grotesque and deformed. Thus, Dr. Jekyll thinks that he can receive the pleasure that both parts of his being crave without each being encumbered by the demands of the other. However, Mr. Hyde evokes feelings of dread and abhorrence in Dr. Jekyll's friends who beseech him to give up his “friendship” with this Edward Hyde. Edward Hyde gradually becomes ever more powerful than his ‘good’ counterpart and ultimately leads Dr. Jekyll to his doom. “Jekyll and Hyde” as an eponymous term has become a synonym for multiple personality in scientific[4] and lay literature[5] and the novel has also been considered a case demonstration of substance dependence.[6]

## DUALISM, RELIGION, AND THE NOVEL

A religion that is dualistic admits not only that the universe comprises good and evil, or light and darkness, but also that though these are eternally opposed they are coeternal, coexistent, and equipotent.[7] This is an important distinction from nondualistic, monistic religions where evil comes about as an accident during creation of the Universe or as a result of powerful beings that can be good or bad as per what serves them or injures them and not because they are evil for the sake of being evil. Here, the good and the evil are often derived from the same source or from one another, much like the *Pandavas* and *Kauravas* in the *Mahabharata*. Zoroastrianism is often cited as an example of a dualistic religion where the concentration of all that is good is around *Ahura Mazda,* and all that is evil around *Ahra Mainyu*. These two forces are at constant war and only at the end will good finally vanquish evil. Interestingly, Christianity, the religion Stevenson was born into, rejects dualism and preaches a monistic origin to the universe from one, infinite, and self-existing spiritual being who freely created everything. However, the dualism of the human soul and the body which it animates was made clearer and is emphasized by the church. In the same vein, Christianity holds that evil is the necessary limitation of finite created beings and is a consequence of creation of beings possessed by free will. As an imperfection inherent in the manufacturing process of individuals, evil is tolerated by God.[1]

In the novel, Stevenson creates a hero in Dr. Jekyll, who aware of the evil in his own being, and sick of the duplicity in his life, succeeds by way of his experiments on himself in freeing the pure evil part of his being as Mr. Hyde, so that each can indulge in a life unfettered by the demands of the other. As Dr. Jekyll says, “With every day and from both sides of my intelligence, the moral and intellectual, I thus drew steadily to that truth by whose partial discovery I have been doomed to such a dreadful shipwreck: that man is not truly one, but truly two.” He further adds,”… that I learned to recognize the thorough and primitive duality of man;… if I could rightly be said to be either, it was only because I was radically both”. Mr. Edward Hyde he describes as, “a second form and countenance substituted, none the less natural to me because they were the expression, and bore the stamp, of lower elements in my soul” and that, “Edward Hyde, alone in the ranks of mankind, was pure evil”.[2] Thus, Stevenson creates in Dr. Jekyll and Mr. Hyde, two equipotent, coexistent, and eternally opposed components that make up a “normal” individual. Here, good and evil are not related but are two independent entities, individuals even, different in mental and physical attributes and constantly at war with each other. Evil now does not require the existence of good to justify itself but it exists simply as itself, depicted as being the more powerful, the more enjoyable of the two, and in the end ultimately it is the one that leads to Dr. Jekyll's downfall and death. This is because Dr. Jekyll in the last phases of his lucidity recognizes the danger that Mr. Hyde poses to society and altruistically decides to do away with himself. Stevenson seems to discard Christian notions of monism and embrace dualism as described above.

The novel needs to be looked at in the context of its setting of Victorian London. Stevenson seems to make a comment not only about the dualism present in every individual but also in society as a whole, where the aristocracy that superficially was genteel and refined, had dark secrets to hide behind the high walls of the mansions in which they lived. Most of the action takes place in the night time and much of it in the poorer districts of London, considered the abode of evil-doers. Most significantly, Mr. Hyde enters and leaves Dr. Jekyll's house through the *back* door which seems a metaphor for the evil that lies behind the façade of civilization and refinement.

## DUALISM, PHILOSOPHY, AND THE NOVEL

Dualism as a philosophy signifies the view that the universe contains two radically different kinds of being or substance-matter and spirit, body, and mind.[7] The ancient Greeks distinguished profoundly the soul and the body as the dictum states: “The body is a tomb.” Evil therefore was a result of an infinite soul trapped in a finite body. Plato for instance was strongly dualistic in that he expressed the view that the soul exists independently of the body. The rational soul is a spiritual substance distinct from the body within which it dwells, much like the chariot and a charioteer.[8] Dualism served a great purpose in the European Renaissance when Descartes described the mind exclusively as a substance that thinks and matter exclusively as an extended substance. This dualism enabled a wholly mathematical science of physics to come about where every fact in the material world was to be explained on basis of measurements. In this scheme, the psyche is immeasurable and thus not open to either understanding or intervention.[1]

In the novel, Stevenson creates a hero who by way of a concoction (that he compares with alcohol in course of the novel) intervenes in his “normal” mental processes and unleashes Mr. Hyde. This new persona not only is pure evil but also has a countenance that suggests “Satan's signature” and a body that is “something troglodytic”.[2] Here, not only the psyche is shown as a process that can be mediated by external tangible methods (the mysterious concoction) but also that a change in the psyche is associated with a change in the body or the soma. Stevenson seems to eschew traditional mind–body dualism to a remarkably modern monistic way of looking at the mind–body functioning.

## ECHOES OF “*THE STRANGE CASE OF DR JEKYLL AND MR. HYDE”* IN FREUDIAN PSYCHODYNAMIC CONCEPTS

The issues raised in the novel find resonance with the Freudian concepts of instincts, life and death instincts, and the structural theory of the mind propounded by Freud.

Freud defined instincts variously but most cogently as “a concept that is on the frontier between the mental and somatic, as the psychical representative of the stimuli originating from and reaching the mind, as a measure of the demands made upon the mind for work in the consequence with its connections with the body.” Freud developed the theory of instincts in relation to the concept of libido and the consequent foundation of the psychosexual phases of development. However, aggression as a component of the libidinal drives became increasingly important and could not be ignored. It was therefore elevated to the status of a separate instinct. It was further realized that humans were neither exclusively nor essentially good. Freud introduced his final theory of life and death instincts in 1920. Freud postulated that the death instinct is a dominant tendency of all organisms and their cells to return to a state of inanimateness. The death instinct represented the aggressive instincts and Freud later separated the libidinal and aggressive instincts from the ego and located them in a vital stratum of the mind which is independent of the ego. This line of thought led to the further differentiation of the psyche as per the “Structural Theory” into the id, ego, and superego.[9]

The characters in the novel manifest characteristics of the structural theory of the mind. Mr. Hyde would seem easily recognizable as the id, seeking instant gratification, having an aggressive instinct, and having no moral or social mores that need be followed. He takes pleasure in violence and similar to the death instinct ultimately leads to his own destruction. Dr. Jekyll is then the ego; he is conscious and rational, and is dominated by social principles. He has a difficult time juggling between the demands of the id, represented by Mr. Hyde, and the superego as represented by the proclaimed and implicit morals of Victorian society which prided itself on refinement and goodness, and is shocked by the seeming nonchalance with which Edward Hyde indulges in his debaucheries. In the novel, Dr. Jekyll gives in to his impulses and after initial pleasure soon cannot control their power. Rather than let Mr. Hyde go free and realizing that Hyde needs Jekyll to exist, he decides to end his own life.

Further, by labeling Mr. Hyde as a “troglodyte”, Stevenson seems to make a comment on the theories of evolution and that he considered Hyde that is savage, uncivilized, and given to passion: poorly evolved. Edward Hyde represents a regression to an earlier, less civilized, and more violent phase of human development.

## A WORK AHEAD OF ITS TIME

*The strange case of Dr. Jekyll and Mr. Hyde* can be seen at various levels. As a story, it talks about the concept of good and evil that exists in all of us. At another level, it is a critique on the hypocrisy and double standards of the society. It is also an interesting study into the mind of the author and into the theories of dualism. Finally, it can be seen as a remarkable study into human psychology that presaged the structural personality theories as detailed by Freud.
